# Are female children more vulnerable to the long-term effects of maternal depression during pregnancy?

**DOI:** 10.1016/j.jad.2015.09.039

**Published:** 2016-01-01

**Authors:** Catherine Quarini, Rebecca M. Pearson, Alan Stein, Paul G. Ramchandani, Glyn Lewis, Jonathan Evans

**Affiliations:** a Warneford Hospital, Warneford Lane, Oxford OX3 7JX, UK; b School of Social and Community Medicine, University of Bristol, Oakfield House, Oakfield Grove, Bristol BS8 2BN, UK; c Department of Psychiatry, University of Oxford, Warneford Lane, Oxford OX3 7JX, UK; d Centre for Mental Health, Imperial College London, Hammersmith Campus, London W12 0NN, UK; e UCL Division of Psychiatry, University College London, Charles Bell house, 67–73 Riding House Street, London W1W 7EJ, UK

**Keywords:** ALSPAC, Gender, Maternal depression, Adolescent depression, Child depression

## Abstract

**Background:**

Female fetuses are more vulnerable to high levels of maternal glucocorticoids. We examined whether exposure to prenatal maternal depression, a condition associated with high glucocorticoids, carries greater risk for depression at 12 and 18 years in girls.

**Methods:**

Our sample comprised 7959 mothers and children from the Avon Longitudinal Study of Parents and Children following imputation for missing data. Maternal depression was assessed pre-and post-natally, and offspring depression at ages 12 and 18. We used logistic regression models to examine the relationship between exposure to prenatal and postnatal depression and offspring depression at 18 and 12 and interactions with gender.

**Results:**

There was an interaction between prenatal depression and gender (*P*=0.027) and between postnatal depression and gender (*P*=0.027) for offspring depression at 18. Following adjustment in pre-natally depressed mothers, the odds ratio for offspring depression at 18 was 1.55 (95% c.i. 1.03–2.34) for girls and 0.54 (0.23–1.26) for boys. In post-natally depressed mothers, the odds ratio for offspring depression at 18 was 1.15 (0.70–1.89) in girls and 3.13 (1.52–6.45) in boys.

However there was no evidence for interaction between prenatal or postnatal depression and gender (*P*=0.559 and 0.780 respectively) for offspring depression at 12.

**Limitations:**

As expected with this large cohort spanning over 18 years, there was loss-to-follow-up.

**Conclusions:**

This is the first evidence in humans that increased vulnerability of female fetuses to maternal stress responses during pregnancy persists into adolescence. One explanation for gender differences emerging later is more depressive symptomatology is attributed to heritable risk at 12, whereas biological processes involved in brain development at 18 may be influenced by foetal programming. If replicated, this study has potential to help understand intergenerational transmission of depression, a leading cause of morbidity worldwide.

## Highlights

• We examined the relationship between maternal prenatal depression and child depression.
• We also investigated the interaction with child gender.
• Depression risk at age 18 following prenatal maternal exposure is greater in girls.
• Depression risk at age 18 following postnatal maternal exposure is greater in boys.
• The impact of maternal stress responses during pregnancy persist into adolescence.

## 1 Introduction

Many adult diseases partly have their origins in utero as a result of foetal programming, the process by which uterine conditions lead to enduring changes in bodily structure or function that may increase risk for chronic conditions in later life. In a now landmark paper, Barker reported that human foetal undernutrition in mid to late gestation can lead to the foetus growing in a disproportionate way and a greater risk of coronary heart disease in adulthood. Babies born at a low birth weight, or who were small in relation to placental size, were at increased risk of coronary heart disease, and the mechanism underlying this is thought to be that maternal undernutrition can slow foetal cell division through altered concentrations of growth factors and hormones, particularly insulin and growth hormone (Barker, 1995). It has been shown in animal models, including primates, that if stress occurs during prenatal brain development this can lead to enduring changes in offspring behaviour, including response to stressors. Prenatal maternal stress leads to the foetal brain being exposed to excess glucocorticoids, which can lead to enduring alteration of the foetal hypothalamo-pituitary-adrenal function in the offspring throughout childhood, adolescence and adult life (Kapoor et al., 2006).

In humans one important cause of an altered stress response is depression. The risk of depression during adolescence is thought to be increased in the offspring of mothers who were depressed during the perinatal period. A study following 151 mother–child dyads from pregnancy to the child’s 16th birthday found that those children exposed to maternal depression during pregnancy were 4 times more likely to become depressed than those children never exposed to maternal depression, and episodes of depression during pregnancy were part of a wider pattern of maternal depression that often began prior to pregnancy and persisted throughout the child’s life (Pawlby et al., 2009). This alone does not tell us whether the increased risk of depression in children is due to genetic inheritance, prenatal foetal exposure, or childhood environment. The mechanism for this intergenerational transmission of risk is not fully understood but there is some evidence that prenatal depression and postnatal depression are independent risk factors for offspring adolescent depression, that act via different pathways. This was demonstrated in a large population cohort study which showed that maternal prenatal depression was associated with child adolescent depression regardless of the mother’s educational level, whereas postnatal maternal depression was only associated with child adolescent depression in mothers with lower educational levels, suggesting that maternal education can moderate the effect of postnatal depression on adolescent mental health, but cannot moderate the pathway for prenatal depression influencing adolescent mental health. As education can influence the impact of postnatal depression, but not of prenatal depression, this suggests that the two act via different pathways, one being modified by education and the other not (Pearson et al., 2013).

The neuroendocrine feedback system between the hypothalamus, pituitary and adrenal glands, can be altered in depression resulting in persistently raised cortisol (Antonijevic, 2008, Min et al., 2012). The effect of this alteration in the maternal stress response during pregnancy on the neural development of the foetus could explain how exposure to prenatal depression increases risk of depression in offspring (Douglas, 2005).

One of the mechanisms by which maternal depression during pregnancy could affect the developing foetus is through altered cortisol transmission across the placenta, and there is accumulating evidence from animal and human studies that this is the case. Maternal prenatal anxiety is associated with the foetus being exposed to higher levels of maternal cortisol when the mother is stressed (O’Donnell et al., 2012). A recent study using the same cohort that we investigate here has shown that maternal prenatal anxiety and depression have an impact on diurnal cortisol variation in adolescent offspring, and the authors suggest that the mechanism for this may be through prenatal programming of the hypothalamopituitary adrenal axis resulting from exposure to high maternal cortisol (O’Donnell et al., 2013).

There is increasing evidence from animal studies that female foetuses are more vulnerable both to high levels of glucocorticoid exposure and prenatal stress than male foetuses (Richardson et al., 2006). Animal studies have shown there are differences in the biology of placental function between male and female foetuses. Female foetuses are more susceptible to anxiety, stress and depression responses in adulthood if they were exposed to maternal prenatal stress, whereas males exposed to prenatal stress are more likely to have learning and memory problems (Glover and Hill, 2012). In mice, more corticosterone crosses from the maternal bloodstream to the placenta in female compared to male foetuses following a stress response in the mother (Montano et al., 1993). If the effect of prenatal depression operates through this mechanism in humans, we would expect to find differences in risk arising from prenatal exposure according to offspring gender.

Understanding the mechanisms of developing depression, particularly in young people, provides an important first step and opportunity for developing methods for prevention. This is an important public health priority as depression is now the leading cause of disability worldwide according to the World Health Organisation (World Health Organisation). Adolescents with depression are at increased risk of depression and anxiety disorders in adulthood, and have increased rates of unemployment, educational underachievement, alcohol abuse, and early parenthood (Fergusson and Woodward, 2002). A large cohort study has demonstrated that frequent depressive episodes in adolescence are followed by poor adult mental health outcomes including major depression, anxiety disorder, suicidal ideation and suicide attempt, as well as greater levels of unemployment and dependence on welfare, even when confounding factors were controlled for (Fergusson et al., 2007).

We have previously demonstrated that both prenatal and postnatal depression is associated with increased risk of depression in offspring at age 18 using data from a large longitudinal UK cohort (Pearson et al., 2013). However, the moderating role of offspring gender was not investigated. Here we investigate whether maternal prenatal depression confers a greater risk of developing adolescent depression on girls than on boys in this cohort. We tested the hypotheses that, at 18 years, girls of prenatally depressed mothers would be more susceptible to depression than boys of prenatally depressed mothers. We also wanted to test children at age 12 to ascertain whether any gender differences were present from early adolescence and persisted into early adulthood, or whether differences emerged during adolescence. We were interested in looking at these two time points, at the beginning and end of adolescence, as different biological processes are involved in brain development (myelination and synaptic pruning) at age 18 compared to age 12, and these processes may be influenced by foetal or early life programming so be more subject to gender influences and timing of maternal depression than earlier in life, when genetic influences alone are likely to be stronger.

We examined evidence for these hypotheses using data from a large longitudinal cohort with 7959 mother–child pairs (after data imputation) followed up until the children were 18 years of age.

## 2 Methods

Our sample comprised participants from the Avon Longitudinal Study of Parents and Children (ALSPAC) which recruited the children of 14,541 pregnancies born between 1990 and 1992 (Fraser et al., 2013). This is an ongoing population-based study which has been running for over 20 years and is investigating the effects of a wide range of variables on the physical and emotional development of children.

Pregnant women living in the area of the former Avon Health Authority in South-West England with an expected delivery date between 1 April 1991 and 31 December 1992 were invited to take part, and the children of 14,541 pregnancies were recruited. Regular objective measurements made in research clinics as well as self-report questionnaires on aspects of health and lifestyle have been collected on the cohort of mothers since early pregnancy and on them and their children since.

Ethical approval for the study was obtained from the ALSPAC Ethics and Law Committee and the Local Research Ethics Committees. After complete description of the study to the subjects, written informed consent was obtained.

### 2.1 Measures

#### 2.1.1 Exposure measure

Maternal depression was measured using the self-report Edinburgh Postnatal Depression Scale (Murray and Carothers, 1987) and a binary variable was created using established thresholds. The EPDS is 10 item self-report questionnaire designed to screen for prenatal and postnatal depression in primary care. Scores of greater than 12 have a high sensitivity (81%) and specificity (96%) in predicting major depressive disorder (Eberhard-Gran et al., 2001). The EPDS questionnaires were sent to pregnant women by post at 18 weeks and 32 weeks of pregnancy and at 8 weeks and 8 months after birth. The average of the 2 prenatal scores was taken as a measure of prenatal depression and the average of the 2 postnatal scores were taken as a measure of postnatal depression. Averaging two repeated measures provides a more stable and reliable estimate of the levels during those periods. From these averaged scores we calculated a binary score based on whether the average score was above or below the established threshold of 13.

#### 2.1.2 Outcome

Our primary analysis focused on a diagnostic measure of depression at age 18. Depression at age 18 was assessed using the Clinical Interview Schedule Revised, with the questions completed by the 18 year old. This has been shown to be a reliable tool for measuring depression when compared to other standardized interviews, and is as accurate when administered by lay interviewers as by psychiatrists (Lewis et al., 1992). The main outcome was meeting ICD-10 criteria for mild, moderate or severe depression. This was our primary outcome because it provides the most validated and clinically relevant estimate of depression.

However, in secondary analysis aiming to compare the gender effects at different ages, we also investigated depressed mood at age 12. At this time point depression was measured at age 12 using the Mood and Feelings Questionnaire-Short Version (MFQ). This rating scale is used in children and adults and asks about how they have been feeling recently, with them self-rating a number of statements which could indicate depression as ‘not true’, ‘sometimes true’ or ‘true’ according to how they felt over the past 2 weeks (http://devepi.duhs.duke.edu/mfq.html). In order to compare whether any differences in patterns of association with the CIS-R at 18 and depressed mood at age 12 were due to the use of different measures we compared associations with another MFQ measure at age 18.

#### 2.1.3 Adjustment

All models were adjusted for maternal educational (highest level achieved), maternal age at birth of the index child and parity. These variables were chosen as previous work shows they are the variables most strongly associated with both maternal and offspring depression. Models are shown with and without mutual adjustment for maternal depression at the other time period (the effect of prenatal depression adjusted for postnatal depression and vice versa).

#### 2.1.4 Missing data

As there was significant loss-to-follow-up as would be expected in a study with such a large sample size and spanning over 20 years, for the main analyses we imputed data using multiple imputation by chained equations (Royston, 2009). The sample size in the imputed models was 7959, this included those with at least one maternal measure of depression and at least one child depression measure. This was particularly important when investigating the interaction with gender as large sample sizes are needed to have sufficient power to provide statistical evidence for interactions. This is a recommended approach for missing data (Sterne, 2009) which allows for uncertainty by creating multiple copies of the dataset (in this case 60) each with missing data replaced by imputed values. Data for those individuals who miss this particular variable is estimated based on others variables that these individuals have completed and the pattern of missing data. In the case of depression status at 18 we used earlier measures of depressed mood and several socio-economic variables which predict whether or not data were missing (Pearson et al., 2013). Further information is available on request. Analyses were also conducted on the sample with complete data on all variables. The sample size varied according to the extent of missing data, for example, for the findings in 18 year olds 1904 girls and 1517 boys provided complete data (see flow chart). In the ALSPAC sample those with missing data are more likely to be male, non-white and eligible for free school meals (indicating economic hardship) (Boyd et al., 2012). The pattern of results were comparable between the complete data set and the data set which also included the imputed values.

## 3 Analysis

We analysed the association between prenatal depression and depression in the child at age 18. We then analysed the association between postnatal depression and depression in the child at age 18. Our results using both imputed and non-imputed data are displayed in Table 1.

Using a logistic regression model for the post imputation sample of 7959 mothers and offspring, we calculated odds ratios for depression in the offspring at age 18 years for mothers with prenatal and postnatal depression and the interactions with gender. We modelled the interactions in a mutually adjusted model where the combined effects of both timings (i.e., those who had depression at both timings, prenatally only or postnatally only) were partialled out and estimates represent the independent effects of each timing. Following the results of this work, we were interested to know whether the patterns observed in terms of gender differences had been apparent since childhood, or only emerged in later adolescence. Therefore, we used data from the same cohort, taken when the children were aged 12, to also analyse the association between maternal prenatal depression and child depression, and maternal postnatal depression and child depression.

We adjusted for maternal education, maternal age, and parity in all models.

## 4 Results

Amongst the 4566 participants who completed the CIS-R, 360 (8%) met ICD-10 criteria for depression at 18. The prevalence in girls was 10.6% and boys 4.3%.

*Main analyses:* Using the post imputation sample of 7959, there was statistical evidence for an interaction between prenatal depression and gender (*P*=0.027) (Table 1). Investigating the association separately by gender (following adjustments), we found that if their mother reached above threshold for depression during pregnancy, the odds ratio for a diagnosis of depression in girls at age 18 was 1.55 (95% C.I. 1.03–2.34), *p* value 0.035. The odds ratio for boys was 0.54 (95% C.I. 0.23–1.26), *p* value 0.155 (Table 1). We also analysed the association between postnatal depression and offspring depression at age 18; there was evidence for an interaction between postnatal depression and gender (*P*=0.01). Examining this separately by gender (following adjustments), for girls the odds ratio of depression at 18 was 1.15 (95% C.I. 0.70–1.89), *p* value 0.555 for boys it was 3.13 (95% C.I. 1.52–6.45), *p* value 0.002.

These results suggest that the risk following prenatal exposure is greatest in girls at age 18 and the risk following postnatal exposure is greatest in boys. The findings using non-imputed data show a similar pattern although the effects are weaker as predicted.

Because we found this gender difference, and there are suggestions that depression appears to show different risk patterns at different ages (Thapar et al., 2012, Goodman et al., 2011) we wanted to know whether this differential gender pattern was observed in early adolescence as well. Depression at age 12 was measured using the MFQ. Using the same sample post imputation of 7959, there was no evidence for an interaction between prenatal maternal depression and gender (*P*=0.599) or between postnatal maternal depression and gender (*P*=0.780) for the outcome of depression in early adolescence (age 12 years). Where mothers were depressed prenatally (following adjustments), the odds ratio for depression in girls at 12–13 years was 1.96 (95% CI 1.30–2.96) *p* value 0.001; for boys at 12–13 years it was 1.62 (95% CI 1.01–2.59) *p* value 0.045. Where mothers were depressed postnatally, the odds ratio for depression in girls at 12–13 years was 1.71 (95% CI 1.06–2.76) *p* value 0.029; for boys at 12–13 years it was 1.53 (95% CI 0.89–2.64) *p* value 0.120 (Table 2).

As depression at age 12 was assessed using the MFQ, a different rating scale to that used at age 18, we compared our findings for 18 year olds obtained using the CIS-R to comparator observations in the same cohort using the MFQ in later adolescence, to ensure that the two rating scales were interchangeable. We found the same pattern of findings for 18 year olds when MFQ scores were used instead of CIS-R scores (where girls were at higher risk from prenatal depression and boys were at higher risk from postnatal depression), the results were statistically weak however and this may be explained by the fact that the MFQ is a less specific measure of depression than the CIS-R Table 3.

Analyses of complete cases with data on all variables for each model showed similar results with evidence that depression at 18 was a greater risk for girls exposed to prenatal depression (OR girls 1.67 95% c.i. 1.07–2.59, *p* value 0.02 c.f. OR boys 0.97 95% c.i. 0.41–2.28, *p* value 0.94) and for boys exposed to postnatal depression (OR girls 1.50, 95% c.i. 0.87–2.61, *p* value 0.15 c.f OR boys 2.54, 95% c.i. 1.22–5.30, *p* value 0.01) (see Supplementary tables for further details).

Finally to check the results were not attributable to exposure to maternal depression at later stages in child’s life, the effect of the number of times the mother was above the threshold for depression from child’s age 1–10 (0–6 times) was investigated. This also associated with risk of CIS-R depression at 18 (OR for each further episode in the mother=1.11, (95% CI 1.02–.20, *p*=0.012)), however there was no evidence for an interaction with child gender (interaction term *p*=0.384).

## 5 Strengths and limitations

As depression during pregnancy is a strong risk factor for depression following childbirth (Nulman et al., 2012) separating out the effects of prenatal and postnatal depression on the child requires a very large sample size, which we were able to achieve. Other strengths include the use of data from a longitudinal cohort study spanning 20 years, rather than using retrospective recall of maternal depression status, and the use of validated scales to measure depression status.

Some limitations of the study should be noted. The study contained some missing data, although our imputations suggested this could not explain the findings. As with all investigations of moderation, statistical power was relatively reduced when investigating interactions between depression and gender. This highlights the importance of the large sample size and the possibilities of robust imputation which is a unique strength of this study. Other limitations inherent in a study such as this one include confounding factors which may have an impact on the development of depression in young people, such as quality of parenting, relapsing and remitting maternal depression not present at the data collection times in our study, other parental mental or physical illness, other people present in the household or influences of other caregivers, household income, and quality of education.

## 6 Discussion

We found that maternal prenatal depression is associated with an increased risk of depression in 18 year old girls compared to 18 year old boys. In contrast we found that maternal postnatal depression is associated with an increased risk of depression in 18 year old boys compared to girls. When we examined the effect on depression at 12 there appeared to be no moderating effect of gender.

One explanation for the gender differences only emerging in later adolescence is that depression in late adolescence is different as different biological systems are developing. A greater portion of the variance is attributed to heritable risk at 12 years old than 18 (Nivard et al., 2015) which does not differ by gender of child or timing of exposure to maternal depression. At 18 different biological processes are involved in brain development (myelination and synaptic pruning) which may be influenced by foetal or early life programming and thus explain why the gender and timing effects are manifest in late adolescence.

Depressive symptomatology, both pre and postnatally, was based on an average score over two time points, in order to obtain a picture of the mother’s mood over a more enduring period of time.

This adds further weight to the suggestion that the effects of prenatal and postnatal depression on offspring operate through different mechanisms (Pearson et al., 2013). This is important as the aetiology of depression is poorly understood despite the fact that it contributes more to the worldwide burden of illness than any other disease, and is often a chronic, lifelong condition.

Our finding that the effect of maternal prenatal depression on late adolescent depression is moderated by the gender of the offspring has implications both for our understanding of the intergenerational transmission of depression, and for our understanding of the origins of late adolescent depression, which often persists into adulthood.

Recent research has shown several sex-specific adaptations of the placenta in humans (Clifton, 2010) and it has been suggested that sex-specific differences in placentae may influence the risk of disease in later life (Gabory et al., 2013). There is increasing evidence that human female foetuses are more vulnerable to maternal stress. Several small studies in humans have found some provisional evidence of gender differences in the effects of prenatal stress exposure. One study found (de Bruijn et al., 2009) that girls born to mothers who experienced depression or anxiety during pregnancy had higher cortisol levels as toddlers (23–60 months), whereas no difference was found in boys. Another study (Van den Bergh et al., 2008) examining the effects of prenatal maternal anxiety on depression in adolescents found that exposed offspring of both genders had abnormal cortisol profiles but only girls experienced more depressive symptoms. A more recent study showed that higher levels of maternal cortisol in earlier gestation was associated with a larger amygdala volume in girls only, and more affective problems in girls (Buss et al., 2012).

Interestingly, the female offspring vulnerability was not replicated when we examined postnatal depression; rather the reverse pattern was seen. The vulnerability of boys to postnatal depression is consistent with research on the disruptive effects of postnatal depression on parenting (Stein et al., 2012) and mother–child interaction (Stein et al., 1991) which may be more harmful for boys than girls (Mensah and Kiernan, 2010). Research has shown that mothers interact differently with boys and girls, for example, in depressed mothers, speech style when talking to their infants differed according to gender (Murray et al., 1993). Postnatal depression in the mother appears to have a particularly adverse effect on the cognitive development of boys (Grace et al., 2003) and this is still apparent in school achievement in adolescence (Murray et al., 2010). Our results suggest that the risk of adolescent depression following exposure to maternal prenatal depression operates through a different pathway than the risk following exposure to maternal postnatal depression. This is consistent with the theory that HPA axis disruption in pregnancy is causally related to offspring depression and has a greater effect on female foetuses compared to males.

**Fig. 1:**
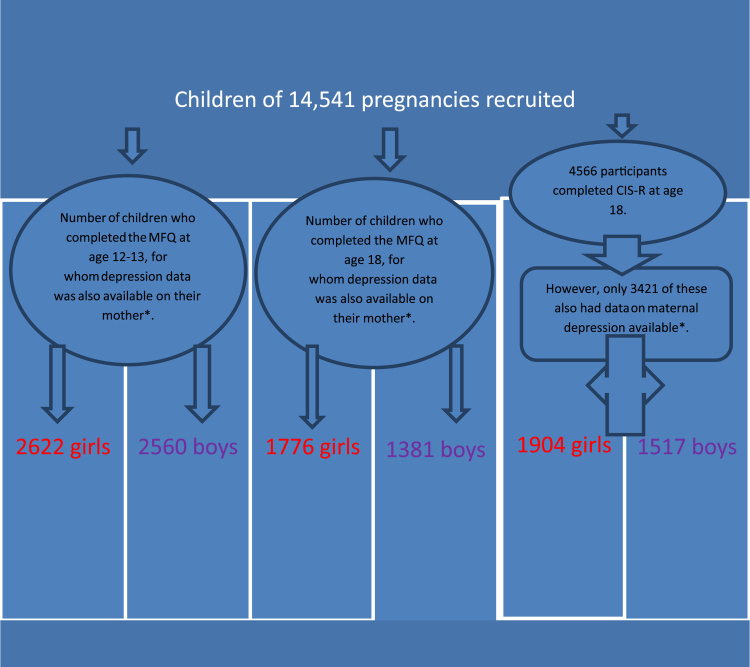
Flow chart showing numbers of participants in each category. *And also data on maternal education, maternal age and parity.

Our finding that maternal prenatal depression is associated with an increased risk of depression in 18 year old girls, but not in 18 year old boys, whereas the opposite pattern is seen with postnatal depression, has the potential to inform our understanding of the possible biological mechanisms involved in adolescent depression. It also suggests the possibility that early sensitive developmental periods may be implicated in adolescent depression (Bornstein, 1989). These data also add weight to the emerging evidence that although prenatal and postnatal depression both confer increased risks of depression in the offspring, they may act via different mechanisms. Overall these findings are important because depression is now the leading cause of disability worldwide (World Health Organisation) Fig. 1.

## Figures and Tables

**Table 1:** Primary analyses showing effects of timing of maternal depression on adolescent offspring depression at age 18 years using the CIS-R, *N*=7959 (imputed sample) mutually adjusted model.

	Odds ratio (95% CI) for a diagnosis of depression in **girls** at age 18 using the CIS-R		Odds ratio (95% CI) for a diagnosis of depression in **boys** at age 18 using the CIS-R		Interaction with gender in mutually adjusted model
	*N*=3860		*N*=4099		
	Unadjusted for other timing^a^	Mutually adjusted^b^	Unadjusted for other timing^a^	Mutually adjusted^b^	
Prenatal depression in the mother (average score >12 as compared to average score <=12)	1.56 (1.07–2.27) *p*=0.020	1.55 (1.03–2.34) *P*=0.035	0.82 (0.40–1.68) *p*=0.591	0.54 (0.23–1.26) *P*=0.155	*P*=0.027
Postnatal depression in the mother average score >12 as compared to average score <=12)	1.41 (0.91–2.20) *p*=0.126	1.15(0.70–1.89) *P*=0.555	2.39 (1.27–4.48) *p*=0.007	3.13 (1.52–6.45) *P*=0.002	*P*=0.027

a Adjusted for maternal education, age and parity only.

b Prenatal depression adjusted for postnatal depression and postnatal depression adjusted for prenatal depression.

**Table 2:** Effects of timing of maternal depression on adolescent offspring depression at age 12 years using the MFQ *N*=7959 (imputed sample) mutually adjusted model.

	Odds ratio for high levels of depressed mood in **girls** at age 12 using the MFQ		Odds ratio for a diagnosis of depressed mood in **boys** at age 12 using the MFQ		Interaction with gender in mutually adjusted model
	*N*=3860		*N*=4099		
	Unadjusted for other timing	Mutually adjusted	Unadjusted for other timing	Mutually adjusted	
Prenatal depression in the mother (average score >12 as compared to average score <=12)	2.2 (1.52–3.17) *P*<0.001	1.96 (1.30–2.96) *P*=0.001	1.76 (1.17–2.65) *p*=0.007	1.62 (1.01–2.59) *P*=0.045	*P*=0.559
Postnatal depression in the mother average score >12 as compared to average score <=12)	2.33 (1.53–3.56) *p*<0.001	1.71 (1.06–2.76) *p*=0.029	1.93 (1.2–3.1) *p*=0.006	1.53 (0.89–2.64) *P*=0.120	*P*=0.780

**Table 3:** Table showing effects of maternal depression on adolescent offspring depression at age 18 years using the MFQ , *N*=7959 (imputed sample).

	Odds ratio for high levels of depressed mood in **girls** at age 18 using the MFQ		Odds ratio for a diagnosis of depressed mood in **boys** at age 18 using the MFQ		Interaction with gender in mutually adjusted model
	*N*=3860		*N*=4099		
	Unadjusted for other timing	Mutually adjusted	Unadjusted for other timing	Mutually adjusted	
Prenatal depression in the mother (average score >12 as compared to average score <=12)	1.55 (1.12–2.12) *p*=0.007	1.40 (1.0–1.95) *P*=0.045	1.01 (0.66–1.57) *p*=0.946	0.90 (0.55–1.46) *p*=0.681	0.161
Postnatal depression in the mother average score >12 as compared to average score <=12)	1.90 (1.33–2.70) *p*<0.001	1.65 (1.13–2.39) *p*=0.009	1.56 (1.01–2.42) *p*=0.048	1.66 (1.02–2.70) *p*=0.043	0.975

## Footnotes

Appendix A Supplementary data associated with this article can be found in the online version at doi:10.1016/j.jad.2015.09.039.
