# Supplementary material for: Are female children more vulnerable to the long-term effects of maternal depression during pregnancy?
Source: J Affect Disord. 2016 Jan 1;189:329–35. doi: 10.1016/j.jad.2015.09.039 (PMC4650986; doi:10.1016/j.jad.2015.09.039)
Supplement: Supplementary file 1 — Supplementary material [file mmc1.pdf]

Supplementary tables

Complete cases CIS-R

	Odds ratio for a diagnosis of depression in girls (non-imputed data)	Odds ratio for a diagnosis of depression in boys (non-imputed data)	Interaction with gender (non-imputed data)
Number of participants	1904	1517	Total n = 3421
Antenatal depression in the mother (average score >12 as compared to average score <=12)	1.67 (95% c.i. 1.07 – 2.59, p value 0.02)	0.97 (95% c.i. 0.41 – 2.28, p value 0.94)	P value 0.08
Postnatal depression in the mother average score >12 as compared to average score <=12)	1.50 (95% c.i. 0.87 – 2.61, p value 0.15)	2.54 (95% c.i. 1.22 – 5.30, p value 0.01)	P value 0.07

Complete cases MFQ at age 12

	Odds ratio for high depressed mood in girls using MFQ at 12	Odds ratio for high depressed mood in boys using MFQ at 12	Interaction with gender
Number of participants	2622	2560	
Antenatal depression in the mother	2.41 (95% c.i. 1.48 – 3.93) P<0.001	2.22 (95% c.i. 1.36 – 3.62) P=0.001	P= 0.543
Postnatal depression in the mother	2.72 (95% c.i. 1.41 – 5.26) P<0.001	3.01 (95% c. i. 1.55 – 5.86) P<0.001	P= 0.639

Complete cases MFQ at age 18

	Odds ratio for high depressed mood in girls	Odds ratio for high depressed mood in boys	Interaction with gender
Number of participants	N =1776	N=1381	
Antenatal depression in the mother	1.55 (95% c.i. 1.04 – 2.30) P=0.031	1.00 (95% c.i. 0.54 – 1.84) P=0.994	P=0.125
Postnatal depression in the mother	1.55 (95% c.i. 0.90 – 2.73) P=0.116	2.18 (95% c. i. 1.55 – 5.86) P= 0.020	P= 0.443
